# Effectiveness of Video Teletherapy in Treating Obsessive-Compulsive Disorder in Children and Adolescents With Exposure and Response Prevention: Retrospective Longitudinal Observational Study

**DOI:** 10.2196/66715

**Published:** 2025-01-27

**Authors:** Jamie D Feusner, Nicholas R Farrell, Mia Nunez, Nicholas Lume, Catherine W MacDonald, Patrick B McGrath, Larry Trusky, Stephen Smith, Andreas Rhode

**Affiliations:** 1 NOCD, Inc Chicago, IL United States; 2 Department of Psychiatry University of Toronto Toronto, ON Canada; 3 Centre for Addiction and Mental Health Toronto, ON Canada; 4 Department of Women's and Children's Health Karolinska Institutet Stockholm Sweden

**Keywords:** digital behavioral health, youth, cognitive-behavioral therapy, exposure and response prevention, CBT, ERP, OCD, psychiatry, clinical trial, psychology, video therapy, teletherapy, e-therapy, e-counseling, cyber-counseling, adolescents, adolescence, obsessive-compulsive disorder, retrospective study, longitudinal study, observational study, ERP therapy

## Abstract

**Background:**

An effective primary treatment for obsessive-compulsive disorder (OCD) in children and adolescents as well as adults is exposure and response prevention (ERP), a form of intervention in the context of cognitive-behavioral therapy. Despite strong evidence supporting the efficacy and effectiveness of ERP from studies in research and real-world settings, its clinical use remains limited. This underuse is often attributed to access barriers such as the scarcity of properly trained therapists, geographical constraints, and costs. Some of these barriers may be addressed with virtual behavioral health, providing ERP for OCD through video teletherapy and supplemented by app-based therapeutic tools and messaging support between sessions. Studies of teletherapy ERP in adults with OCD have shown benefits in research and real-world settings in both small and large samples. However, studies of teletherapy ERP in children and adolescents thus far have been in small samples and limited to research rather than real-world settings.

**Objective:**

This study reports on the real-world effectiveness of teletherapy ERP for OCD in the largest sample (N=2173) of child and adolescent patients to date.

**Methods:**

Children and adolescents with OCD were treated with live, face-to-face video teletherapy sessions, with parent or caregiver involvement, using ERP. Assessments were conducted at baseline, after 7-11 weeks, and after 13-17 weeks. Additionally, longitudinal assessments of OCD symptoms were performed at weeks 18-30, 31-42, and 43-54. We analyzed longitudinal outcomes of OCD symptoms, depression, anxiety, and stress using linear mixed models.

**Results:**

Treatment resulted in a median 38.46% (IQR 12.50%-64.00%) decrease in OCD symptoms at 13-17 weeks, and 53.4% of youth met full response criteria at this point. Improvements were observed in all categories of starting symptom severity: mild (median 40.3%, IQR 8.5%-79.8%), moderate (median 38.4%, IQR 13.3%-63.6%), and severe (median 34.1%, IQR 6.6%-58.5%). In addition, there were significant reductions in the severity of depression, anxiety, and stress symptoms. The median amount of therapist involvement was 13 (IQR 10.0-16.0) appointments and 11.5 (IQR 9.0-15.0) hours. Further, symptom improvements were maintained or improved upon in the longitudinal assessment periods of weeks 18-30, 31-42, and 43-54.

**Conclusions:**

These results show that remote ERP treatment, assisted by technology, can effectively improve both core OCD and related depression, anxiety, and stress symptoms in children and adolescents with OCD in a real-world setting. Notable outcomes were achieved in a relatively small amount of therapist time, demonstrating its efficiency. Demonstrating the usefulness of a delivery format that overcomes several traditional barriers to treatment, these findings have implications for widespread dissemination of accessible, evidence-based care for children and adolescents with OCD.

## Introduction

Obsessive-compulsive disorder (OCD) is a common and often disabling mental health condition that affects 0.25%-4% of children and adolescents [1-3]. Without treatment, OCD can persist into adulthood. Further, it can significantly interfere with a young person's development, education, and relationships. Fortunately, OCD in young people can be effectively managed through psychotherapy, medication, or a combination of the two. Exposure and response prevention (ERP), a form of intervention in the context of cognitive-behavioral therapy (CBT), is particularly effective for treating OCD in children and adolescents. It has been extensively tested in clinical trials [4-6] and is recommended as the first-line treatment for OCD [4,7-10].

However, accessing ERP can be challenging due to a shortage of therapists trained in this specialized technique, along with the costs and geographical limitations of attending in-person therapy sessions [11-13]. Therapists with adequate training in ERP for children and adolescents are even more scarce [14,15]. Remotely delivered ERP, for example, therapy that delivers ERP via the phone or video, can potentially overcome many of these obstacles by increasing access to specialized providers, reducing travel time and costs for families, and enabling therapists to work with patients in their natural environments that trigger OCD symptoms.

Meta-analyses of remotely delivered CBT with ERP for OCD have shown benefits in studies for adults and children or adolescents [16-18]. The largest meta-analysis (22 studies; N=1796) that included studies of both adults and children or adolescents (9 studies) found that remote ERP resulted in superior reduction of OCD symptoms compared with control conditions (Hedges *g*=0.94, 95% CI 0.60-1.27; *P*<.001), and there was no significant difference in efficacy compared with in-person CBT or ERP (*g*=–0.104, 95% CI –0.391 to 0.184; *P*=.479) [17]. The majority of these studies have included mostly self-referred participants, those with any severity of OCD, concurrent psychiatric medications, and comorbidities such as depression and anxiety disorders. Further, in several countries, remote ERP, delivered both with and without therapist support, has been implemented in routine care and found to be safe and efficacious, with medium to large effect sizes [19-21]. Remote video telemedicine treatment, in general, has shown noninferiority to traditional in-person treatment for OCD, anxiety, and depression in a head-to-head study in adults [22]. Non-inferiority of remote CBT delivered by telephone compared with face-to-face CBT has also specifically been demonstrated in adolescents with OCD [23]. Remote treatments have the added benefit of allowing therapy to take place in the patient's everyday environment. This can be especially helpful for younger patients, as it enables therapists to work with them in the settings that trigger their OCD symptoms, such as at home or school.

The convenience of teletherapy, coupled with the widespread ownership of smartphones, makes this form of treatment a promising option for families seeking help for OCD. NOCD is a virtual behavioral health provider that specializes in the delivery of evidence-based treatments, including ERP, for obsessive-compulsive and related disorders. NOCD has created a virtual therapy program that delivers ERP through video teletherapy. The program aims to maximize therapeutic impact while minimizing the time required from therapists by eliminating travel time and allowing for more efficient, remote delivery sessions. The program uses treatment design elements from an open clinical trial in adults that demonstrated the effectiveness of combining the NOCD app with face-to-face therapy sessions in reducing OCD symptoms significantly and efficiently while achieving good patient satisfaction [24]. To extend the reach of its treatment and enhance its effectiveness, NOCD provides one-on-one video sessions with a therapist, along with additional support through messaging with their therapist, a web-based OCD community, and peer support. We previously reported clinical outcomes from 3552 adults with OCD treated with ERP [25]. In this retrospective observational study, the median improvement was a 45% reduction in OCD symptoms. Further, 62.9% met the criteria for a full response.

The treatment for children and adolescents at NOCD follows a similar structure as for adults, although it includes involvement of parents and other caregivers. This comprehensive model aims to improve access to effective OCD treatment for children and adolescents, thereby addressing a significant gap in mental health services for young people. The goal of this retrospective observational longitudinal study was to determine clinical outcomes in a large naturalistic sample of children and adolescents with a primary diagnosis of OCD who received ERP treatment via video teletherapy.

## Methods

### Research Design

This was a retrospective, observational longitudinal analysis of clinical data from children and adolescents with a primary OCD diagnosis who received ERP treatment at NOCD from July 23, 2020 (when NOCD began offering child and adolescent treatment), to May 3, 2024, when we froze the data for analysis.

### Population and Sample

From the total pool of child and adolescent patients who received treatment at NOCD between July 2020 and May 2024, our analytical sample included 2173 patients who met the following criteria: (1) primary diagnosis of OCD according to *DSM-5* (*Diagnostic and Statistical Manual of Mental Disorders* [5th Edition]) criteria and Diagnostic Interview for Anxiety, Mood, and OCD and Related Neuropsychiatric Disorders (DIAMOND) assessment, (2) completion of baseline Dimensional Obsessive-Compulsive Scale (DOCS) assessment, and (3) completion of at least one DOCS assessment during weeks 13-17 of treatment.

As part of the general clinical enrollment at NOCD, parents or legal guardians initially reached out to the intake team as self-referrals or received a referral from their insurance or medical provider. Therapists trained by NOCD in OCD assessment and treatment conducted the initial diagnostic evaluations. These evaluations occurred over the first 2 sessions and included a thorough clinical review covering the biopsychosocial aspects of the individual's history and a semi-structured diagnostic interview using the DIAMOND [26]. Those diagnosed with OCD as their primary concern, according to *DSM-5* criteria [27] and the DIAMOND, received treatment. The majority of candidates with an “extreme” rating on the DIAMOND clinician-rated severity scale were directed to more intensive treatment options, such as intensive outpatient programs, partial hospitalization programs, or residential treatment programs. Exceptions to referring patients with an “extreme” DIAMOND rating to more intensive treatment were made on a case-by-case basis, based on clinician judgment. Factors considered included, for example, the patient's level of motivation to engage in outpatient ERP treatment, the presence of comorbidities that might interfere with treatment, as well as the availability and involvement of supportive family members or caregivers who could assist with the treatment process. Referrals for other significant psychiatric or substance use issues, if they were deemed to potentially interfere with ERP treatment, were also made as needed (eg, to child and adolescent psychiatrists or other specialty providers). NOCD generally provides services to individuals aged 5 years and older, although exceptions were made for some 4-year-old patients.

### Treatment Approach

The treatment plan included weekly or twice-weekly 60-minute ERP sessions via video for the first 3 weeks for most, followed by, typically, 10-14 weeks of weekly 60-minute sessions to support the continuation of ERP exercises. During this phase, some transitioned to 30-minute check-in sessions based on their clinical progress. The decision to transition patients to 30-minute check-in sessions was based on clinical judgment considering factors such as symptom reduction, demonstrated mastery of ERP principles, and consistent engagement with between-session exposures. Therapists, while aiming to adhere to this structured approach, could adjust the number or frequency of sessions to meet clinical needs and accommodate patients’ and their parents’ or caregivers’ schedules. Family therapy sessions were scheduled when the therapist deemed them necessary. Treatment duration, number of visits completed, and total therapist contact hours were obtained for each patient. Additionally, patients and their parents or caregivers could engage in asynchronous in-app messaging with their therapists for guidance on homework assignments or for support. App usage (number of app opens) and messaging activity between therapists and patients were tracked to measure treatment engagement. NOCD also offered continuously available support through online monitored community groups. Patients and their parents or caregivers also had access to the NOCD app for tools to set up ERP exercises with their therapist, record the results of their ERP exercises, and access and interact with the online NOCD community. The NOCD app’s ERP tools include ways to set up exposures with instructions, images, scripts, audio recordings, and external links; means to record obsessions and track distress reduction associated with exposure exercises (with graphical depictions); and response prevention tips (see Multimedia Appendix 1 for example screenshots of the NOCD app's key features). Parents or caregivers and caregivers of youth with OCD were able to access weekly support groups facilitated by a trained clinician to receive ongoing support and guidance in reducing accommodation of OCD in the home. Lastly, parents or caregivers received personalized psychoeducation on OCD symptoms in youth and on the maintaining role of familial accommodation and were given guidance in developing a plan to progressively reduce and eventually eliminate any accommodation behaviors.

### Technology Platform

All sessions used a secure, US HIPAA (Health Insurance Portability and Accountability Act)-compliant (and compliant with other countries’ health information privacy regulations) version of Zoom, accessible via personal computing or mobile devices, with live technical support available during business hours to address connectivity issues.

### Therapist Qualifications and Training

Study therapists held master’s or doctoral degrees and were licensed in the states of patients to whom they provided treatment or were associate clinicians who were supervised by licensed therapists. Therapists underwent comprehensive ERP training from NOCD. This included multiple days of focused instruction on OCD and ERP techniques. This was followed by evaluations, including written tests and mock diagnostics, education, and ERP treatment sessions. Therapists were required to pass these written and practical evaluations before starting to treat patients. Additionally, clinicians received further training and guided practice opportunities in the following areas: (1) providing psychoeducation to youth using age-appropriate language and examples, (2) tailoring ERP for OCD in youth, and (3) addressing familial accommodation. Ongoing consultation was provided, including weekly group sessions and periodic case reviews.

### Data Collection and Assessment Instruments

Patients completed self-report assessments to avoid therapist bias. These included the DOCS, which was the primary OCD outcome measure, and the Depression, Anxiety, and Stress Scale (DASS-21) to measure commonly occurring comorbid depressive, anxiety, and stress symptoms. Links to these assessments were sent to patients or parents or caregivers via the NOCD app every 3 weeks. Assessments were completed collaboratively between the patient and their parent or caregiver. Therapists also completed the DIAMOND severity scale, a clinician-rated measure of OCD severity.

#### DOCS

The DOCS [28] is a 20-item self-report measure of OCD symptom severity across 4 domains: contamination, responsibility for harm or mistakes, unacceptable thoughts, and incompleteness or symmetry. Respondents rate the severity of their symptoms on a scale from 0 (no symptoms) to 4 (extreme symptoms) across 5 items: time occupied, avoidance behavior, associated distress, functional interference, and difficulty disregarding obsessions or refraining from compulsions. The 4 subscale scores (range 0-20) can be summed to produce a total DOCS score (range 0-80). A DOCS total score of 18 or above optimally distinguishes someone with OCD from someone without a psychiatric diagnosis [29]. The DOCS has shown good psychometric properties, including strong convergent validity with the Yale-Brown Obsessive Compulsive Scale (*r*=0.54) and the Obsessive-Compulsive Inventory-Revised (*r*=0.69), and is sensitive to the effects of treatment. Further, it shows strong correlations with the Obsessive-Compulsive Inventory-Children’s Version Revised (OCI-CV-R) [30] at baseline (*r*=0.77), at 3 weeks (*r*=0.80), and at 9 weeks (*r*=0.80) of treatment [31]. For the purposes of this analysis, a full response was defined as a ≥35% reduction in DOCS scores and a partial response as a 25%-35% reduction, aligned with recommended criteria for treatment response and remission in OCD [29,31].

#### DIAMOND

The DIAMOND severity scale [26] is a 2-item clinician-rated assessment of the overall severity of an individual’s emotional distress and functional impairment related to OCD symptoms. The clinician makes separate ratings of an individual’s emotional distress and functional impairment on a scale ranging from 1 (normal) to 7 (extreme), and the higher of the 2 ratings is taken as the total severity score. On the DIAMOND clinician-rated severity scale, a score of 7 indicates an “extreme” level of OCD symptoms and functional impairment.

#### DASS-21

The DASS-21 [32] is a 21-item self-report measure of symptoms of depression, anxiety, and stress. It has been widely used in previous research and has consistently shown good psychometric qualities.

### Statistical Analysis

Data, pseudoanonymized before analysis, were examined using a linear mixed model approach, with time points as fixed factors and patients as random factors. This statistical method allowed us to include all available data points while accounting for the repeated measures structure of the data. The time points for rating scale scores included ratings at baseline, the most recent rating obtained between weeks 7-11, and the most recent rating obtained between weeks 13-17. The primary measurement of interest was DOCS score changes from baseline to week 13-17. Since there was some degree of flexibility in the treatment, not everyone had rating scales done at precisely the same session or week of their treatment. Thus, these windows allowed us to measure symptom improvement at approximately a midpoint in treatment (weeks 7-11) and at the end of the active treatment period (weeks 13-17). We only included patients who had data from at least both of these time points. Of the patients who completed both baseline and week 13-17 assessments, a subset also completed assessments during weeks 7-11.

The primary (DOCS scores) and secondary (DASS-21 depression, anxiety, and stress scores) outcome measures were analyzed using linear mixed models, with time points as fixed factors and patients as random factors and with statistical significance set at an α of 0.05. We calculated effect sizes using Hedges *g*. Descriptive statistics, including treatment duration and mean and median symptom improvements, were calculated for those who had a baseline and at least one subsequent rating at 13-17 weeks. The primary analysis included data from both children and adolescents together, with subsequent post hoc analyses examining the effect of age group (children: ages 4-12 years, and adolescents: ages 13-17 years). The model included time (assessment_bin), age group, and their interaction as fixed effects. Similarly, we analyzed the effect of racial and ethnic category with a model including time (assessment_bin), racial and ethnic category, and their interaction as fixed effects. We also analyzed the effect of therapy over time on individual OCD domains, with the respective DOCS subscore as the outcome variable. The model included time (assessment_bin), OCD subtype (subscore_name), and their interaction as fixed effects. A random intercept for each user accounted for individual variability. Analyses were conducted in R.

### Ethical Considerations

The analyses in this study did not require research ethics board review, as this does not meet the criteria for human subject research as defined by federal regulations for human subject protections, 45 CFR 46.102(e); this is a secondary analysis of de-identified data from clinical records, obtained and analyzed retrospectively, and was not the result of a research intervention or interaction. The University of California, Los Angeles (UCLA) institutional review board (IRB) office confirmed that this study did not meet the criteria for human subject research (PRE#20-008583) and thus did not require approval. Compliance with data protection laws was ensured through NOCD’s privacy policy, which all patients agreed to, outlining data use and protection measures.

The UCLA IRB determined that analyses conducted in this study did not meet criteria for human subject research (PRE#20-008583) as defined by federal regulations for human subject protections, 45 CFR 46.102(e). This determination was made because this is a secondary analysis of de-identified data from clinical records, obtained and analyzed retrospectively, and was not the result of a research intervention or interaction. Regarding informed consent, since this was a secondary analysis of existing clinical data, additional research consent was not required beyond the original consent provided for receiving clinical care. All patients agreed to NOCD's privacy policy and terms of service during their clinical care, which included provisions for the use of deidentified data for research purposes. Patients had the ability to opt out of data sharing. Data privacy and confidentiality were maintained through comprehensive de-identification procedures. All direct identifiers were removed from the dataset before analysis. The de-identified data was stored and analyzed on secure, HIPAA-compliant servers with restricted access limited to authorized clinical and research personnel. No direct participant compensation was provided for this retrospective analysis, as it did not involve any additional participation beyond standard clinical care.

## Results

Table 1 summarizes the demographic and psychometric characteristics of the study participants.

**Table 1:** Demographics and psychometrics, N=2173^a^.

Characteristics			Values
Age, mean (SD)			13.44 (2.77)
**Gender, n (%)**			
	Male	1036 (47.68)	
	Female	763 (35.11)	
	Not reported or prefer not to say	374 (17.21)	
**Race and ethnicity, n (%)**			
	White	949 (43.67)	
	Not recorded	721 (33.18)	
	More than one race	177 (8.15)	
	Hispanic or Latino	126 (5.80)	
	Asian, Asian American, or Pacific Islander	95 (4.37)	
	Undisclosed	42 (1.93)	
	Other	40 (1.84)	
	Black or African American	22 (1.01)	
	American Indian or Alaska Native	1 (0.05)	
Insurance pay, n (%)			1102 (50.71)
Cash pay, n (%)			1071 (49.29)
**Medication, n (%)**			
	Currently taking	1058 (48.69)	
	Not currently taking	1115 (51.31)	
DOCS^b^ (baseline), mean (SD)			28.16 (13.50)
**DASS-21^c^ (baseline), mean (SD)**			
	Anxiety	10.96 (8.58)	
	Depression	11.71 (10.17)	
	Stress	17.58 (9.02)	
**Psychiatric medication, n (%)^d^**			
	SSRI^e^	1270 (58.44)	
	ADHD^f^ medication^g^	232 (10.68)	
	Antianxiety or sedative-hypnotic^h^	124 (5.70)	
	Antipsychotic^i^	109 (5.02)	
	Other antidepressant^j^	59 (2.72)	
	SNRI^k^	49 (2.25)	
	Anticonvulsant or mood stabilizer^l^	33 (1.52)	
	TCA^m^	30 (1.38)	
**Comorbid diagnoses, n (%)^d^**			
	Anxiety disorders	569 (26.18)	
	Obsessive-compulsive and related disorders	448 (20.62)	
	Mood disorders	339 (15.60)	
	ADHD	228 (10.49)	
	Tic or Tourette’s disorder	202 (9.30)	
	Autism spectrum disorder	64 (2.95)	
	Trauma and stress-related disorders	42 (1.93)	
	Substance use disorders	2 (0.09)	
	Other	492 (22.64)	
	At least one comorbidity	1200 (55.22)	
	Single comorbidity	704 (32.40)	
	Multiple comorbidities	496 (22.83)	

^a^This sample includes individuals with DOCS assessments available at least at baseline and at 13-17 weeks.

^b^DOCS: Dimensional Obsessive-Compulsive Scale.

^c^DASS-21: Depression Anxiety and Stress Scale-21.

^d^As some individuals were on more than one psychiatric medication and had more than one comorbidity, the total across categories is greater than 100%.

^e^SSRI: selective serotonin reuptake inhibitor.

^f^ADHD: attention-deficit and hyperactivity disorder.

^g^ADHD medications included stimulants (eg, methylphenidate, amphetamine derivatives), non-stimulants (eg, atomoxetine), and alpha-2 adrenergic agonists (eg, clonidine and guanfacine).

^h^Anti-anxiety or sedative-hypnotics included non-benzodiazepine anti-anxiety agents (eg, hydroxyzine, and buspirone), benzodiazepines (eg, clonazepam and lorazepam), and beta blockers (eg, propranolol).

^i^Antipsychotics included both typical and atypical antipsychotics (eg, aripiprazole, quetiapine, risperidone, and ziprasidone).

^j^Other antidepressants included norepinephrine-dopamine reuptake inhibitors (eg, bupropion), serotonin antagonist and reuptake inhibitors (eg, trazodone), and tetracyclic antidepressants (eg, mirtazapine).

^k^SNRI: serotonin–norepinephrine reuptake inhibitor.

^l^Anticonvulsant or mood stabilizers included anticonvulsants (eg, lamotrigine, gabapentin, and oxcarbazepine) and non-anticonvulsant mood stabilizers (eg, lithium).

^m^TCA: tricyclic antidepressant (eg, clomipramine).

### Sample

**Figure 1:**
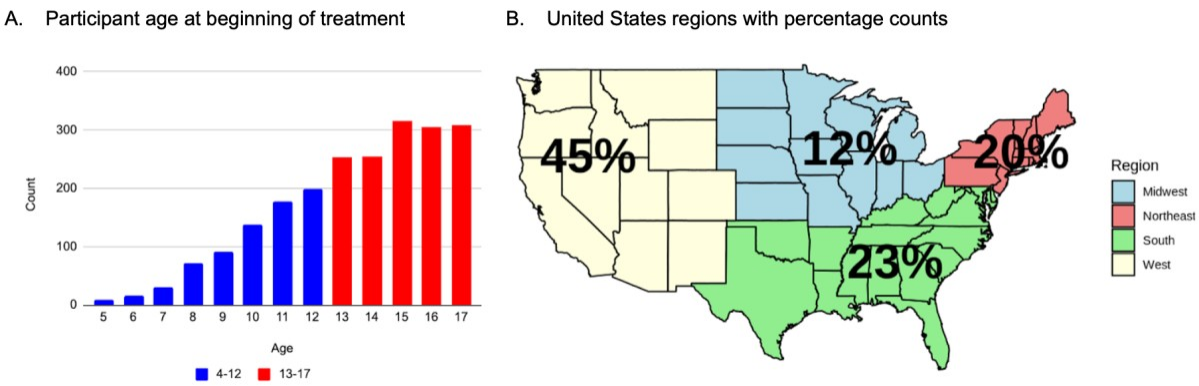
Age and US geographic distributions for the total sample. (A) Participants' age at the beginning of treatment. Blue indicates children, and red indicates adolescents. (B) Percentages of patients by US geographic location of residence.

The total sample size of individuals who had at least a baseline and weeks 13-17 DOCS completed was 2173. The number of individuals who had a baseline, weeks 7-11, and weeks 13-17 DOCS completed was 1797. As shown in Figure 1A, the age distribution of participants at treatment initiation included both children and adolescents. The sample represented diverse geographic regions across the United States (Figure 1B).

### App Use and Messaging

In terms of app usage, 2129 (97.98%) opened the app more than 10 times. In total, 2127 (97.88%) had at least one chat message with their therapist, while 1835 (84.45%) had 10 or more chat messages. The mean number of chat messages for those who had at least one message was 53.53 (SD 87).

### Treatment Duration

The mean treatment duration was 15.12 (SD 7.96, median 15, IQR 1-.86-17.00, mode 16) weeks, the mean number of visits was 13.70 (SD 5.83, median 13, IQR 10.00-16.00, mode 13), and the mean number of therapist hours was 12.60 (SD 5.58, median 11.50, IQR 9.00-15.00, mode 10.50).

### OCD Symptom Results

**Figure 2:**
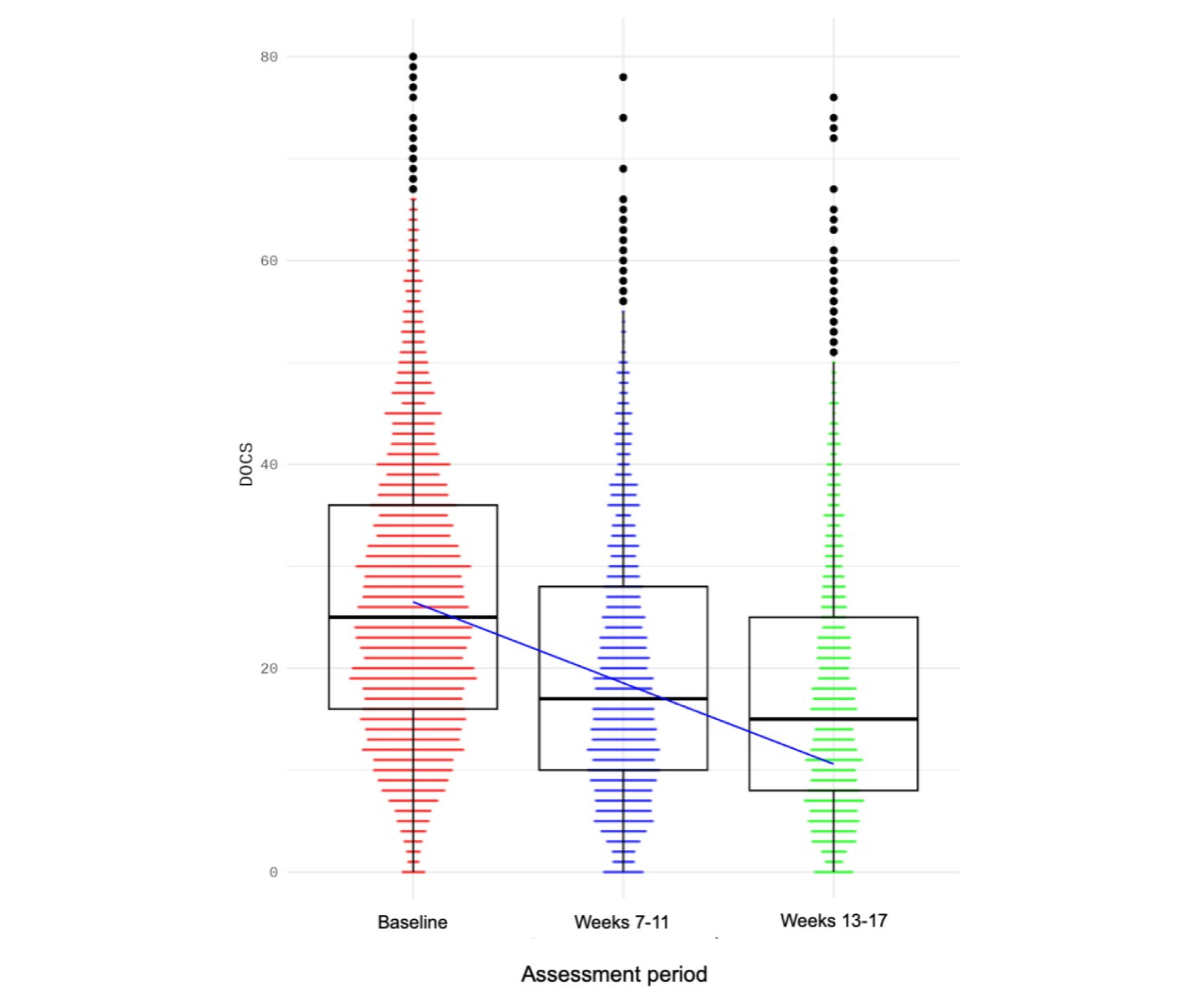
Changes in obsessive-compulsive disorder symptoms as assessed by the Dimensional Obsessive-Compulsive Scale (DOCS) with treatment. Median and interquartile ranges are indicated in the box-and-whisker plots. *P*<.001 for the effect of assessment period.

NOCD treatment resulted in a significant decrease in patient-rated OCD symptoms over time (DOCS scores; effect of time: *F*_1,6927.7_=2785, *P*<.001; *t*_6927.69_=–52.77, *P*<.001; initial to endpoint Hedges *g*=–0.65: “medium” effect size, 95% CI –0.59 to –0.70). From baseline to week 7-11, DOCS scores decreased from a mean of 28.16 (SD 13.50) to a mean of 20.58 (SD 13.00), representing a mean –7.7 (95% CI –7.1 to –8.2) point decrease (27.3%) among participants who completed the forms at the midpoint. By week 13-17, DOCS scores improved to a mean of 17.94 (SD 12.9), representing a mean –10.22 (95% CI –9.7 to –10.7) point decrease (38.4%; see Figure 2 and Table 2). On the individual patient level, median DOCS score improvement was 38.46% (IQR 12.5%-64%).

Further, 54.0% had a ≥35% reduction in OCD symptoms and were categorized as full “responders” [33]. A total of 64.6% achieved either partial (25%-35% reduction) or full response.

An additional analysis using the assessment time period as a categorical fixed effect (rather than ordinal, as was done in the main analysis) confirmed the overall effect of duration on scores (*F*_2,6545.5_=1610.4, *P*<.001). Specifically, there was a significant decrease in scores between baseline and week 7-11 of 7.58 points (SE .179, *t*_6805_=44.707, *P*<.001). There was an additional significant decrease in scores between weeks 7-11 and weeks 13-17 of 1.94 points (SE .224, *t*_6310_=8.674, *P*<.001). Thus, longer treatment duration is associated with continued significant improvements in OCD symptoms.

**Table 2:** Clinical assessments by treatment time point.

Outcome scale and assessment time point			Valid, n	Missing, n			Mean	SD
				Total missing	Did not complete form	Left treatment		
**DOCS^a^**								
	Baseline		2173	0	0	0	28.16	13.50
	Weeks 7-11		1797	376^b^	376	0	20.58	13.00
	Weeks 13-17		2173	0	0	0	17.94	12.90
	Weeks 18-30		1779	394	67	327	15.26	12.01
	Weeks 31-42		1257	916	67	849	14.04	11.71
	Weeks 43-54		859	1314	0	1314	13.51	11.87
**DASS-21^c^ depression**								
	Baseline		2173	0	—^d^	—	11.71	10.17
	Weeks 7-11		1527	646^b^	—	—	9.00	9.38
	Weeks 13-17		1766	407	—	—	8.04	9.19
**DASS-21 anxiety**								
	Baseline		2173	0	—	—	10.96	8.58
	Weeks 7-11		1527	646^b^	—	—	7.94	7.63
	Weeks 13-17		1766	407	—	—	6.93	7.18
**DASS-21 stress**								
	Baseline		2173	0	—	—	17.58	9.02
	Weeks 7-11		1527	646^b^	—	—	13.65	8.79
	Weeks 13-17		1766	407	—	—	12.60	8.73

^a^DOCS: Dimensional Obsessive-Compulsive Scale.

^b^Missing data at weeks 7-11 for DOCS and DASS-21 scales were due to the patient not completing the rating scale(s). Missing data for DOCS at weeks 18-30, 31-42, or 43-54 were due to either the patient not completing the rating scale or the patient having left treatment.

^c^DASS-21: Depression Anxiety and Stress Scale-21.

^d^Not applicable.

### Post Hoc Analysis of Treatment Outcomes by Age Group and Racial and Ethnic Categories

Adolescents had higher baseline scores compared to children (β=4.52, *t*_6045.16_=11.09, *P*<.001). However, the interaction between time and age group was not significant (β=–0.21, *t*_5127.48_=–0.80, *P*=.42), indicating that the rate of improvement over time was not significantly different between the 2 age groups. An additional analysis of responses in children (ages 4-12 years) and adolescents (ages 13-17 years), separately, revealed similar results: in children, the effect of time on DOCS scores: β=–5.10, *t*_2132.88_=–27.06, *P*<.001; in adolescents, the effect of time on DOCS scores (β=–5.39, *t*_4071.89_=–40.99, *P*<.001).

An analysis testing the effects of racial and ethnic categories did not reveal any significant effects of these categories on the overall outcomes (all *P* values≥.10).

### OCD Subtype Results

Treatment resulted in a significant decrease in patient-rated OCD symptoms (DOCS subscores) over time. With the “contamination” subtype taken as the reference group, the reported main effect of time was significant (*F*_1,52,742_=2213.70, *t*_48,510_=–16.31, *P*<.001). A significant main effect of OCD subtype (*F*_3,45,585_=564.98, *P*<.001) indicated that baseline symptom severity differed across subtypes. Patients with the “thoughts” subtype had the highest baseline scores, followed by “responsibility,” “symmetry,” and “contamination.”

The interaction between time and subtype was significant (*F*_3,45,585_=68.85, *P*<.001), indicating that the rate of symptom improvement varied by subtype. The “thoughts” subtype showed the steepest decline in symptoms over time, changing by –1.85 points per assessment time point window (*t*_48,510_=–13.81, *P*<.001), followed by “responsibility” (–1.49 points per assessment time point window, *t*_48,510_=–8.77, *P*<.001) and “symmetry” (–1.20 points per assessment time point window, *t*_48,510_=–4.80, *P*<.001). The “contamination” subtype, serving as the reference group, experienced the slowest decline (–0.86 points per assessment time point window, *t*_48,510_=–16.31, *P*<.001). These results suggest that NOCD treatment is effective across all OCD subtypes, with different magnitudes of improvement over time.

### Depression, Anxiety, and Stress Results

**Figure 3:**
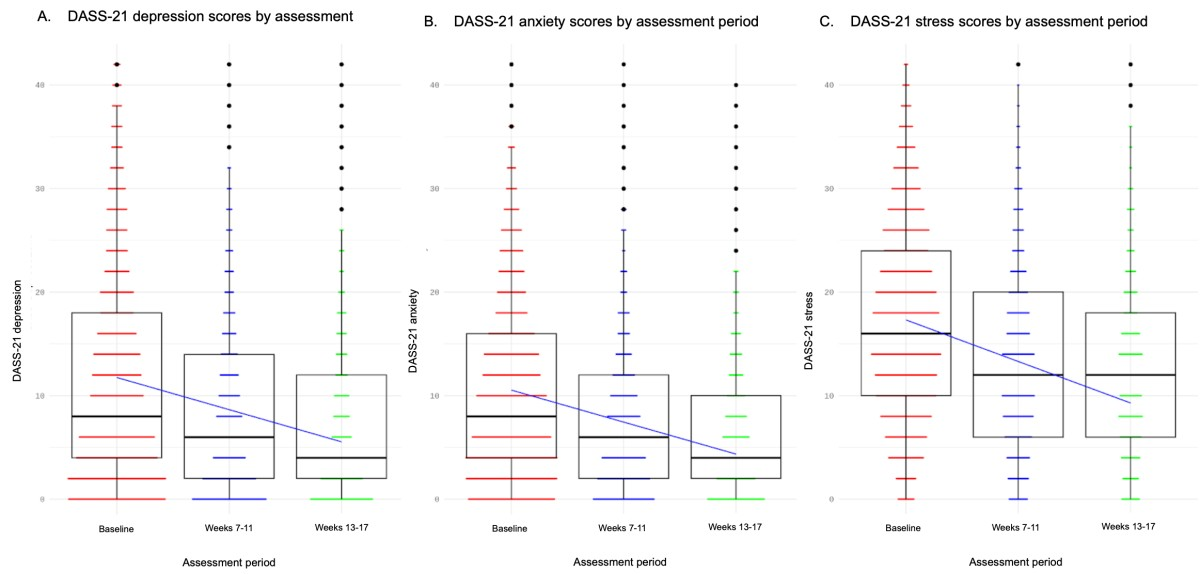
Changes in (A) depression, (B) anxiety, and (C) stress symptoms with treatment, as assessed by the Depression Anxiety and Stress Scale (DASS-21). Median and interquartile ranges are indicated in the box-and-whisker plots. *P*<.001 for the effect of assessment period.

Treatment resulted in significant improvements on the DASS-21 depression (*t*_6415.03_=–30.55, *P*<.001; initial to endpoint Hedges *g*=–.37, 95% CI –0.32 to –0.42), DASS-21 anxiety (*t*_6571.91_=–34.05, *P*<.001; initial to endpoint Hedges *g*=–.43, 95% CI –0.38 to –0.49), DASS-21 stress (*F*_7123.66_=–36.66, *P*<.001; initial to endpoint Hedges *g*=–.52, 95% CI –0.47 to –0.57; Figure 3 and Table 2).

### Post Hoc Analysis of Outcomes Stratified by Starting Clinician-Rated Severity Level

To determine how treatment response differed by different initial severity levels of OCD, we used the DIAMOND clinician-rated severity scale at the initial assessment to stratify patients into 3 groups of severity ratings: “Mild” (severity score of 2 or 3), “Moderate” (severity score of 4 or 5), or “Severe” (severity score of 6 or 7) (n=4 had missing DIAMOND data). For DOCS scores, on the individual patient level, the mild group (n=264) had a median 40.31% (IQR 8.51%-79.80%) reduction, the moderate group (n=1761) a median 38.36% (IQR 13.33%-63.64%) reduction, and the severe group (n=144) a median 34.07% (IQR 6.56%-58.52%) reduction. Response rates from the DOCS were 55.3% for mild, 54.23% for moderate, and 49.30% for severe. A chi-square test showed that these response rates did not differ significantly between severity groups (*χ*²_2_=1.49, *P*=.47), suggesting that treatment was similarly effective regardless of initial severity level.

### Longitudinal Follow-Up

**Figure 4:**
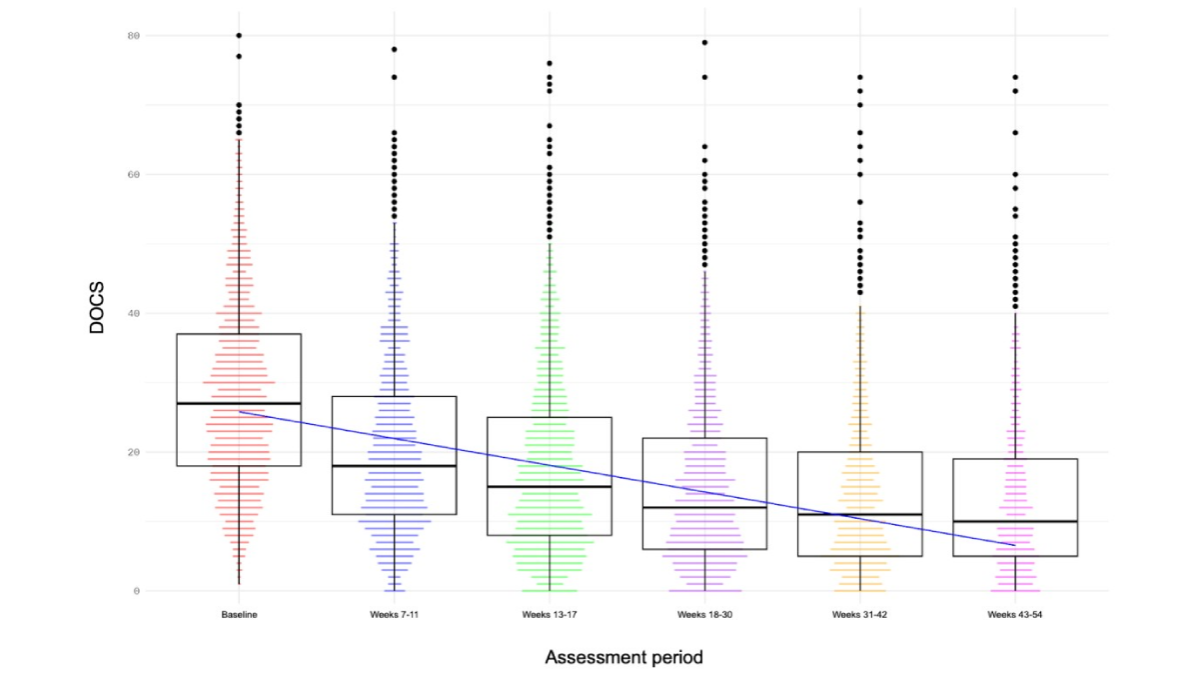
Longitudinal follow-up of obsessive-compulsive disorder symptoms as assessed by the Dimensional Obsessive-Compulsive Scale (DOCS). Median and interquartile ranges are indicated in the box-and-whisker plots. *P*<.001 for the effect of assessment period.

We conducted an analysis of longitudinal follow-up of OCD symptom scores after the active treatment period (weeks 13-17). At the follow-up windows of weeks 18-30, 31-42, and 43-54, most patients maintained their gains or made further improvements (effect of time: *t*_7970.47_=–61.99, *P*<.001; Figure 4 and Table 2). From baseline to week 18-30 (n=1779), DOCS scores decreased from a mean of 28.16 (SD 13.50) to a mean of 15.26 (SD 12.01), representing a mean –12.9 (95% CI –12.11 to –13.7) point decrease (45.81%; Hedges *g*=–1, 95% CI –0.94 to –1.07). By weeks 31-42 (n=1257), DOCS scores improved to a mean of 14.04 (SD 11.71), representing a mean –14.12 (95% CI –13.27 to –14.99) point decrease (50.14%; Hedges *g*=–1.09, 95% CI –1.02 to –1.17). By weeks 43-54 (n=859), DOCS scores improved to a mean of 13.51 (SD 11.87), representing a mean –14.65 (95% CI –13.68 to –15.63) point decrease (52.02%) from baseline (Hedges *g*=–1.12, 95% CI –1.04 to –1.2).

## Discussion

### Principal Findings

The aim of this retrospective observational longitudinal study was to assess the clinical outcomes of a large, naturalistic sample of children and adolescents with a primary diagnosis of OCD who received ERP treatment using video teletherapy. Children and adolescents exhibited significant reductions in their symptoms, demonstrating the effectiveness of this approach for younger populations. Specifically, symptom reduction was notable with a median 38.5% reduction, 54.0% of participants achieving a full response, and 64.6% showing either a partial of full response. The treatment also led to improvements in commonly co-occurring symptoms such as depression, anxiety, and stress symptoms from baseline to end point (weeks 13-17).

There were similar outcomes for children as for adolescents, and outcomes did not significantly differ by racial and ethnic categories. Moreover, the results were largely maintained, or improved upon, for those in long-term follow-up. These outcomes underscore the potential of targeted OCD treatment to alleviate a range of severe and distressing symptoms that stem from their primary OCD diathesis. This is particularly important given that an estimated 50%-87.5% of OCD cases onset before the age of 21 [34,35], and OCD is a chronic condition if left untreated. Further, individuals often endure symptoms for an average of 11 years before receiving treatment [36].

The results highlight the substantial impact and efficiency of this treatment model for OCD and associated symptoms, offering both time and cost savings. The rapid timeframe of these improvements, achieved in a median of 13 sessions and 11.5 hours, represents a significant reduction in both therapist time and treatment duration compared to what has been observed in treatment-as-usual outpatient CBT (37.0, SD 45.0 sessions) [37]. This efficiency has implications for considerable cost reductions for families and insurance providers.

### Comparison to Prior Work

Meta-analyses of remotely delivered CBT with ERP for OCD have consistently shown benefits across studies of both adults, adolescents, and children. The earliest meta-analysis [16] (N=420) found that technology-delivered CBT demonstrated a moderate effect size (d=0.82) for OCD symptom reduction compared to control conditions (although this analysis primarily focused on adults, without specifying results for children or adolescents). A subsequent meta-analysis [18] (N=823) similarly reported a large effect size (*g*=1.17) for remote treatment of OCD symptoms compared to control, including both children and adults in their analysis but without segregating effect sizes by age group. The most recent and largest meta-analysis [17] (N=1796) found that remotely delivered CBT resulted in significant improvement in OCD symptoms compared to control conditions (*g*=0.936) and showed no significant difference in efficacy compared to face-to-face CBT (*g*=–0.104). Notably, this analysis included 9 studies specifically examining children and adolescents, representing 36.8% of the total sample. A pilot study published after these meta-analyses, focusing specifically on video-based CBT for pediatric OCD [38], demonstrated promising results, with a 90% response rate in a small sample (N=29) of children and adolescents. While encouraging, this high response rate may reflect the carefully controlled conditions typical of pilot investigations. Additionally, Weidle's study used clinician-rated outcomes rather than patient-reported metrics.

Importantly, to date, no other studies have examined the real-world effectiveness of video teletherapy for treating OCD in children and adolescents. The current retrospective observational analysis represents the largest naturalistic examination of this treatment approach, with over 2000 patients receiving care within a community-based online clinical practice. Comparison of these results with results from controlled studies is hindered by multiple factors, including differences in sample size, self-selection (in controlled studies), treatment protocols, and outcome measures used, which may have contributed to differences in response rates. These data were from patient-reported OCD symptom scale (DOCS) rather than clinician-rated assessments, are retrospective or observational rather than a prospective study, and have a longitudinal design to evaluate the durability of treatment gains. Despite these methodological distinctions, the findings from our observational analysis, together with findings from previous research studies, suggest video-based delivery of exposure-based therapy is both effective and efficacious for treating OCD in younger populations. Our large-scale, real-world investigation provides promising evidence for the viability of this innovative service delivery model.

The treatment methodology for children and adolescents, as for adults, was influenced by a previously developed and evaluated approach to provide evidence-based ERP treatment efficiently in terms of therapist time [24]. The symptom reduction achieved in this study aligns with that found in earlier research, although direct comparisons are somewhat limited due to differences in setting (real-world clinical versus controlled research environments) and outcome measures used (patient-rated DOCS versus clinician-rated Yale-Brown Obsessive-Compulsive Scale) [36]. Additionally, the use of face-to-face teletherapy distinguishes this treatment from in-person methods previously studied.

In terms of comparison with previously-reported adult outcomes [25], the results in this child and adolescent cohort show a slightly lower magnitude of symptom reduction (median 7.7% lower). This may be due to several factors. Children and adolescents may not as readily comprehend the rationale behind ERP, which might result in some resistance in completing all required homework. In general, the idea of intentionally experiencing distress in the interest of overcoming symptoms may be highly counterintuitive, especially for children. The concepts of habituation with repeated exposures and interruption of compulsions as a way to break the cycle of obsessions and compulsions are abstract concepts that may exceed the cognitive developmental capacities of some. These potential barriers are partially mitigated, however, by parental psychoeducation and their involvement in the treatment.

Further, in this cohort, symptom improvements were relatively consistent across mild, moderate, and severe cases. This indicates the treatment model's broad applicability and effectiveness across different severity levels of OCD, even in those with severe OCD, who achieved a median of 34.07% symptom reduction. This finding emphasizes the treatment's capacity to address the needs of a diverse group of young patients in a time-efficient manner.

The treatment also resulted in decreases in depression, anxiety, and stress symptoms from baseline to endpoint (weeks 13-17). This was unsurprising as, for many with OCD, these symptoms are secondary to their core OCD symptoms; thus, as the OCD symptoms improve, improvements in the secondary symptoms often follow. This can occur to a certain extent even without specific treatments directed at them.

An innovative aspect of the NOCD model was the inclusion of additional patient support mechanisms, such as between-session SMS messaging with therapists, which was used by about 98% of members and in those who used it at an average of about 54 times. Also available was 24-hour access to NOCD’s online support community. It is possible that this might facilitate a sense of belonging and understanding among participants and their parents or caregivers and help normalize their experiences by connecting them with peers facing similar challenges. This peer support, especially from individuals who had successfully completed the NOCD treatment, likely encouraged ongoing engagement and adherence to the therapy process, which is critical given the inherently challenging nature of ERP.

The use of technology, including video teletherapy and integrated communication tools, was pivotal in engaging and effectively treating a wide demographic of young patients across various locations. These technological solutions allowed for the execution of in-session, in vivo exercises tailored to the individual's symptoms and environments, enhancing the relevance and impact of the therapy. Previous research supports the efficacy of remote therapy [17], and the significant symptom improvement rates observed in this study further validate the effectiveness of virtual ERP, comparable to traditional in-person therapy.

### Strengths and Limitations

There are several limitations to this analysis that should be considered. First, the use of the DOCS as the primary outcome measure is a limitation, as it was not designed specifically for use in children and adolescents. Nevertheless, we found it to be correlated with a validated self-report child-specific measure (the OCI-CV-R) [30]. However, because we did not use a rating scale that is widely used in children (such as the Children’s Yale-Brown Obsessive Compulsive Scale (CY-BOCS) [39], direct comparison of results to prior studies that used other scales to assess OCD symptom severity and treatment response in pediatric populations is hindered. In future work, it would be beneficial to incorporate additional parent- or caregiver-completed assessments, such as the CY-BOCS Parent Version [40]. Second, there was a high percentage of missing data for the DOCS for weeks 13-17 and in the longitudinal follow-up periods and for the DASS-21 for weeks 7-11 and 13-17. Using linear mixed models for analyses allowed us to include all available data points (without having to discard all of a patient’s data if, for example, a midpoint assessment was missing). However, the extent of missing data limits our ability to estimate responses for OCD symptoms at midpoint (weeks 13-17), in the follow-up period, and secondary outcomes of depression, anxiety, and stress. Third, this was an observational study design, which inherently precludes making causal inferences about the specific effects of the video teletherapy intervention since there was not a control arm. Relatedly, there were no standardized treatment fidelity checks, typically found in controlled trials, although NOCD therapists received the same standardized training and were audited for adherence. Finally, while we collected satisfaction ratings after sessions, the response rate was low, limiting our ability to comprehensively assess families' satisfaction with the treatment approach. Despite these limitations, the flexibility allowed within the treatment model provides real-world applicability and potential for adaptation to the highly varied individual patient and parent needs. Additionally, the large sample size and longitudinal design offer substantial evidence for the effectiveness of this innovative service delivery approach in a naturalistic clinical setting.

### Conclusions

Overall, ERP delivered via technology-assisted video teletherapy results in clinically significant improvements in OCD, depression, anxiety, and stress symptoms for children and adolescents with OCD. This is achieved in approximately 65% fewer sessions than treatment-as-usual outpatient CBT with ERP. This could translate to lower costs than such lower-intensity approaches and may also translate to reduced costs compared with higher-intensity programs for those with severe symptoms. Further, it is effective for moderate and severe OCD, which for some may prevent the need for higher levels of care such as intensive outpatient, partial hospitalization, residential, or inpatient treatment. Because OCD in this population intimately involves the family, healthier children and adolescents could translate to less stress and better health in family members. In sum, this treatment modality offers a scalable, effective option for accessing evidence-based care, potentially reducing the burden of OCD on young individuals and their families while also presenting an opportunity for significant cost savings compared to traditional treatment methods.

## Appendix

Multimedia Appendix 1 Supplementary Figures. Screenshots demonstrating key features of the NOCD app interface used during treatment, including the home screen, fear hierarchy building tools, exposure exercise support features, and crisis management resources.

## Abbreviations

CBT: cognitive-behavioral therapy
CY-BOCS: Children’s Yale-Brown Obsessive Compulsive Scale
DASS-21: Depression, Anxiety, and Stress Scales
DIAMOND: Diagnostic Interview for Anxiety, Mood, and OCD and Related Neuropsychiatric Disorders
DOCS: Dimensional Obsessive-Compulsive Scale
DSM-5: Diagnostic and Statistical Manual of Mental Disorders (5th Edition)
ERP: exposure and response prevention
HIPAA: US Health Insurance Portability and Accountability Act
IRB: institutional review board
OCD: obsessive-compulsive disorder
OCI-CV-R: Obsessive-Compulsive Inventory-Children’s Version Revised
UCLA: University of California, Los Angeles

## Data Availability

The datasets analyzed during this study are proprietary business assets of NOCD Inc, and are not publicly available. The data may be available from the corresponding author on reasonable request with appropriate data use agreements in place.
