# Supplementary material for: Effectiveness of Video Teletherapy in Treating Obsessive-Compulsive Disorder in Children and Adolescents With Exposure and Response Prevention: Retrospective Longitudinal Observational Study
Source: J Med Internet Res. 2025 Jan 27;27:e66715. doi: 10.2196/66715 (PMC11811664; doi:10.2196/66715)
Supplement: Multimedia Appendix 1 [file jmir_v27i1e66715_app1.docx]

**Supplementary Information**

**Effectiveness of Video Teletherapy in Treating Child and Adolescent Obsessive-Compulsive Disorder with Exposure and Response Prevention: a Retrospective Longitudinal Observational Study**

Authors:

Jamie D. Feusner^1-4^, Nicholas R. Farrell^1^, Mia Nuñez^1^, Nicholas Lume^1^, Catherine W. MacDonald^1^, Patrick B. McGrath^1^, Larry Trusky^1^, Stephen Smith^1^, Andreas Rhode^1^

^1^NOCD, Inc., Chicago, IL, 60601, USA

^2^Department of Psychiatry, University of Toronto, Toronto, ON, Canada

^3^Centre for Addiction and Mental Health, Toronto, ON, Canada

^4^Department of Women’s and Children’s Health, Karolinska Institutet, Stockholm, Sweden

1. Supplementary Figures: Example App Screenshots


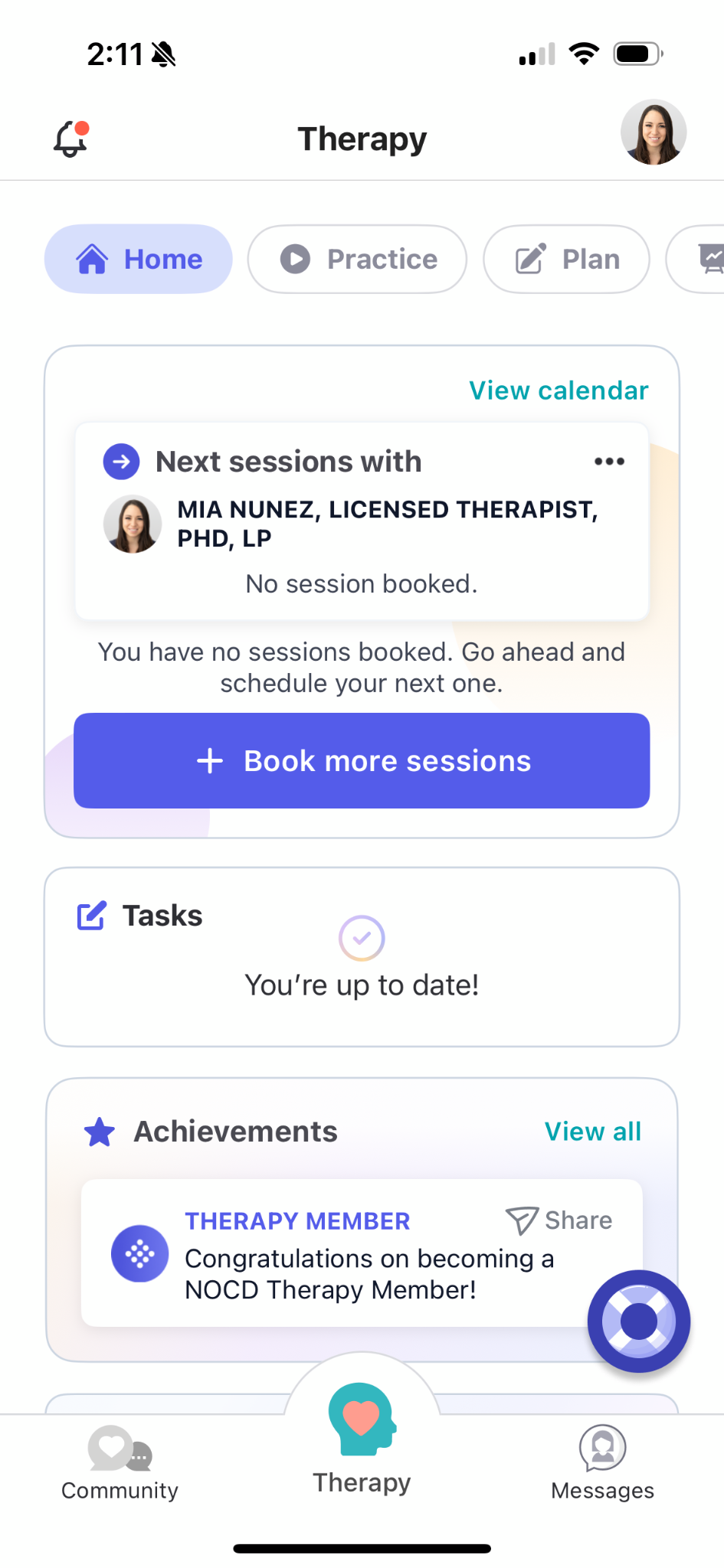


Fig. 1. NOCD App homescreen


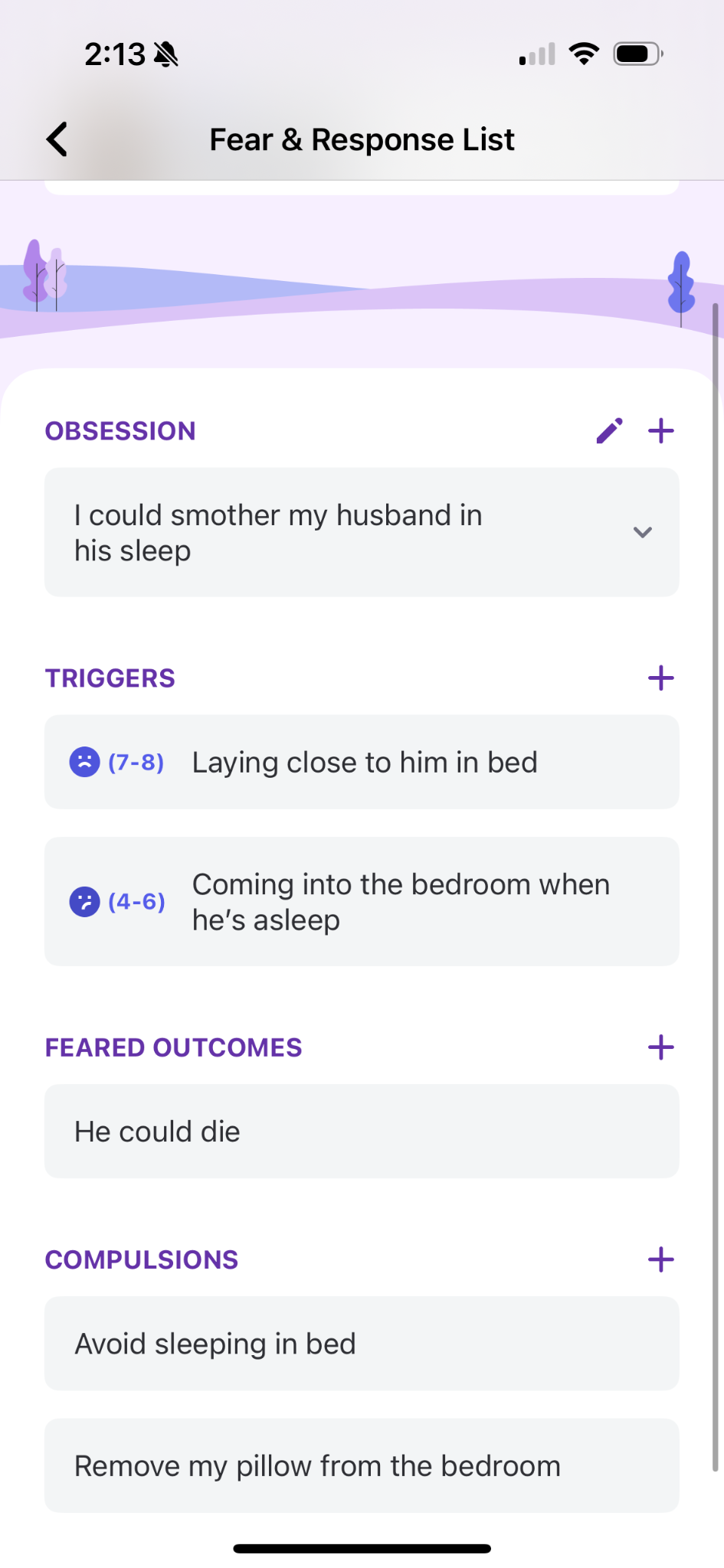


Fig. 2. NOCD App self-monitoring and hierarchy building tool, called “Fears and Response List”


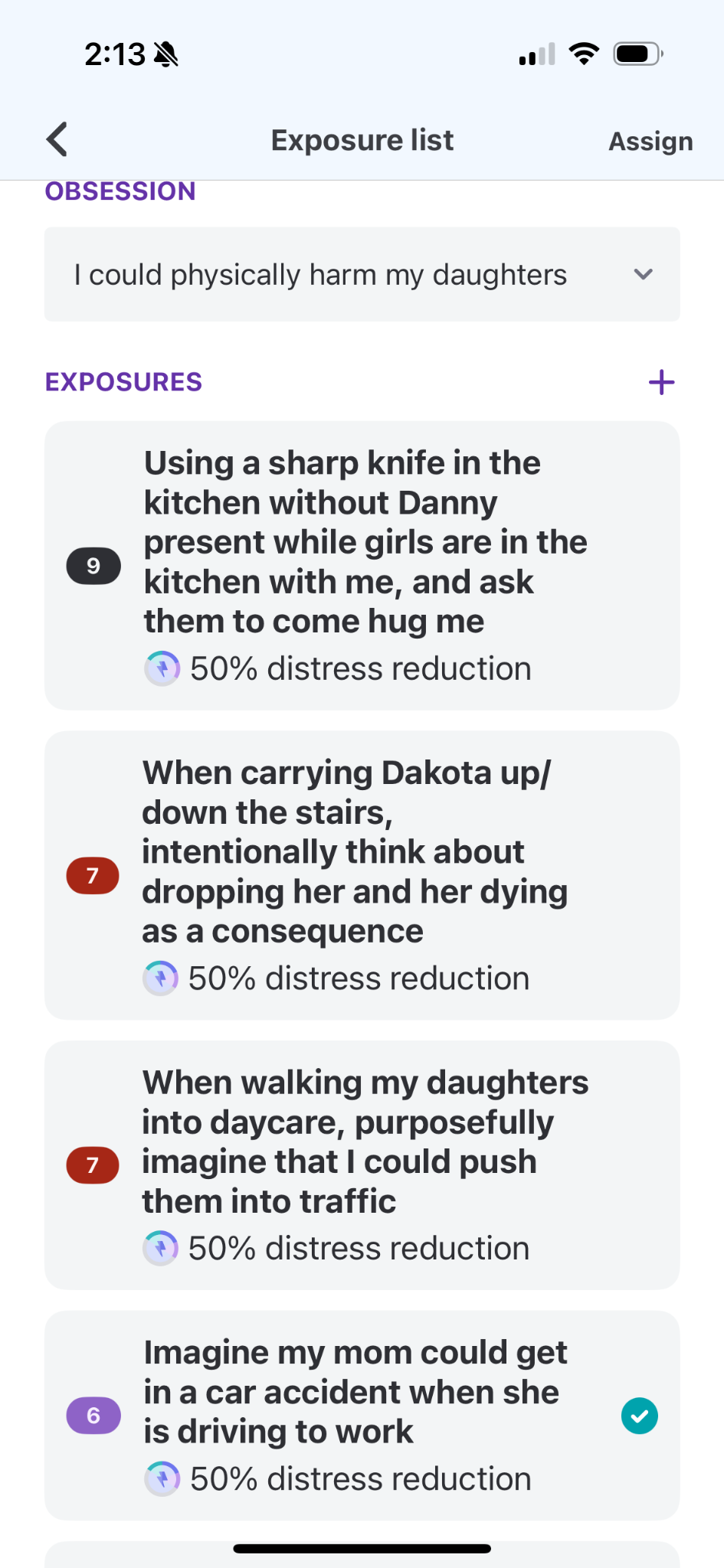


Fig. 3. NOCD App hierarchy


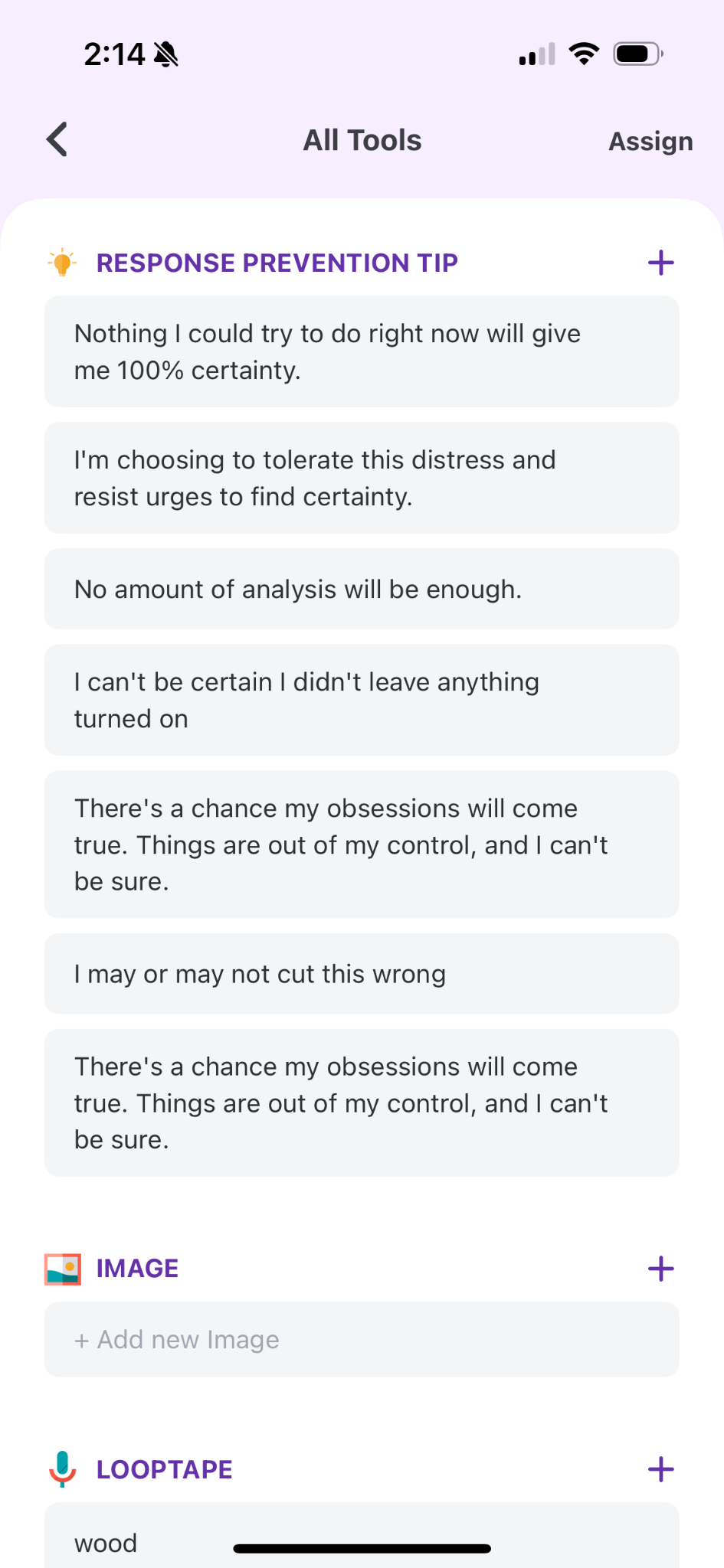

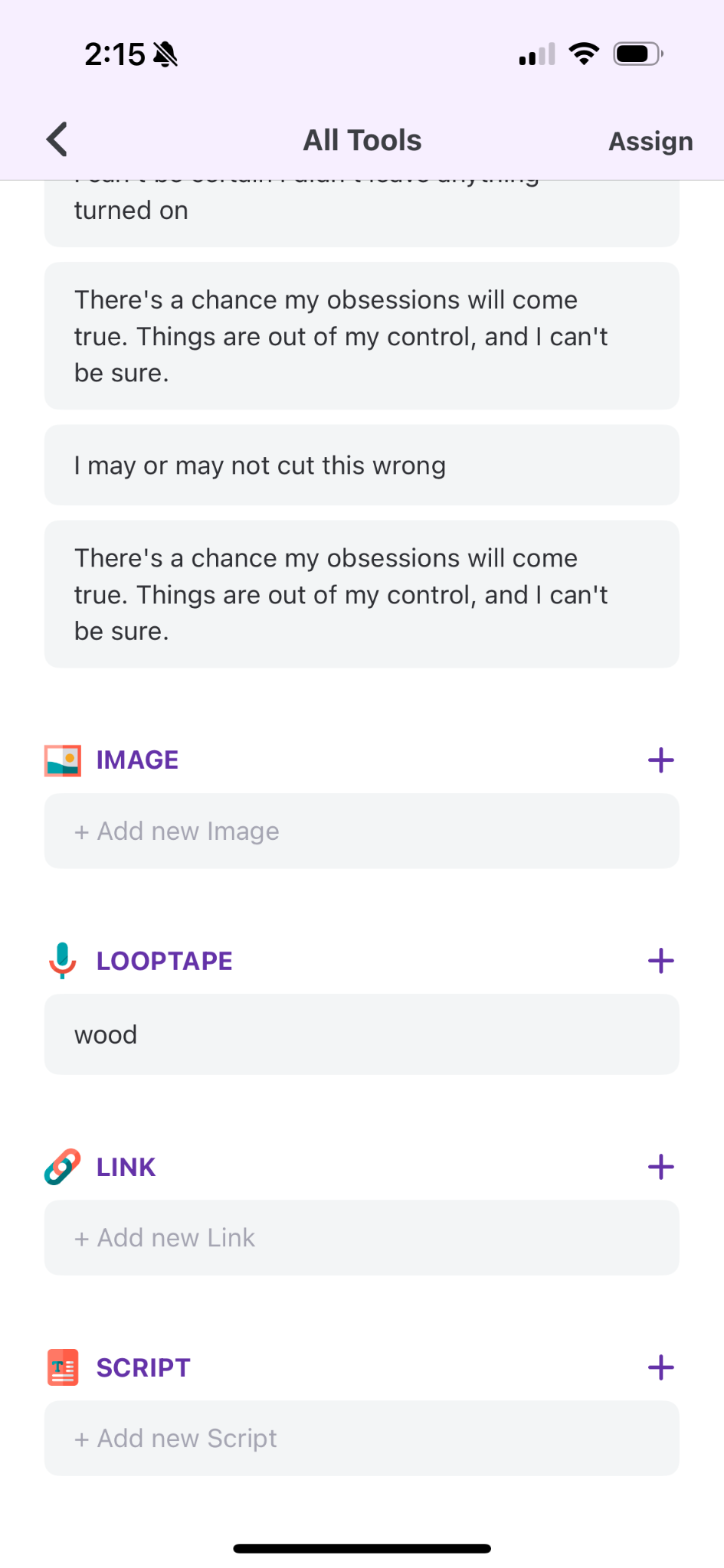


Fig. 4. NOCD App Tools; Response prevention tips and functionality to for therapist or Member to upload images, looptapes, links, and scripts for exposures.


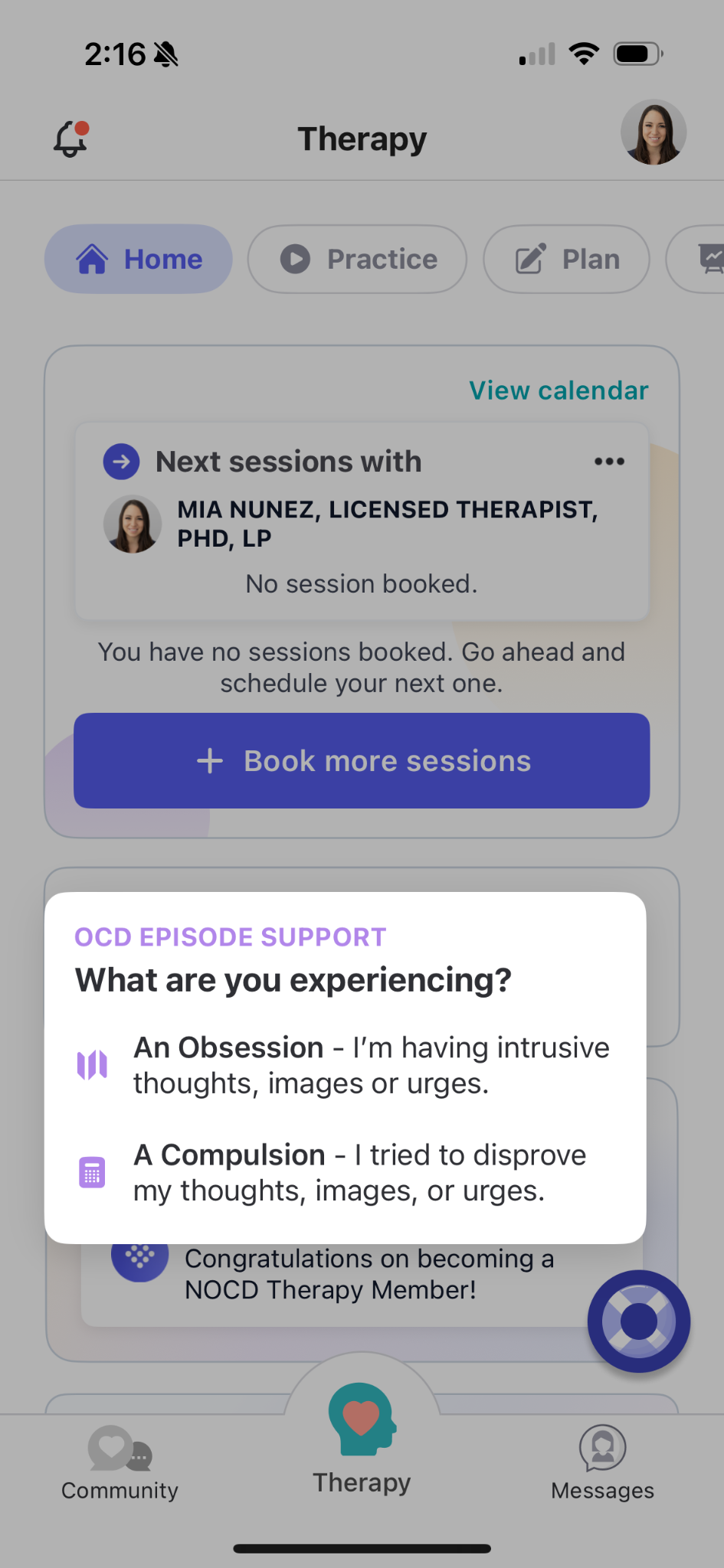

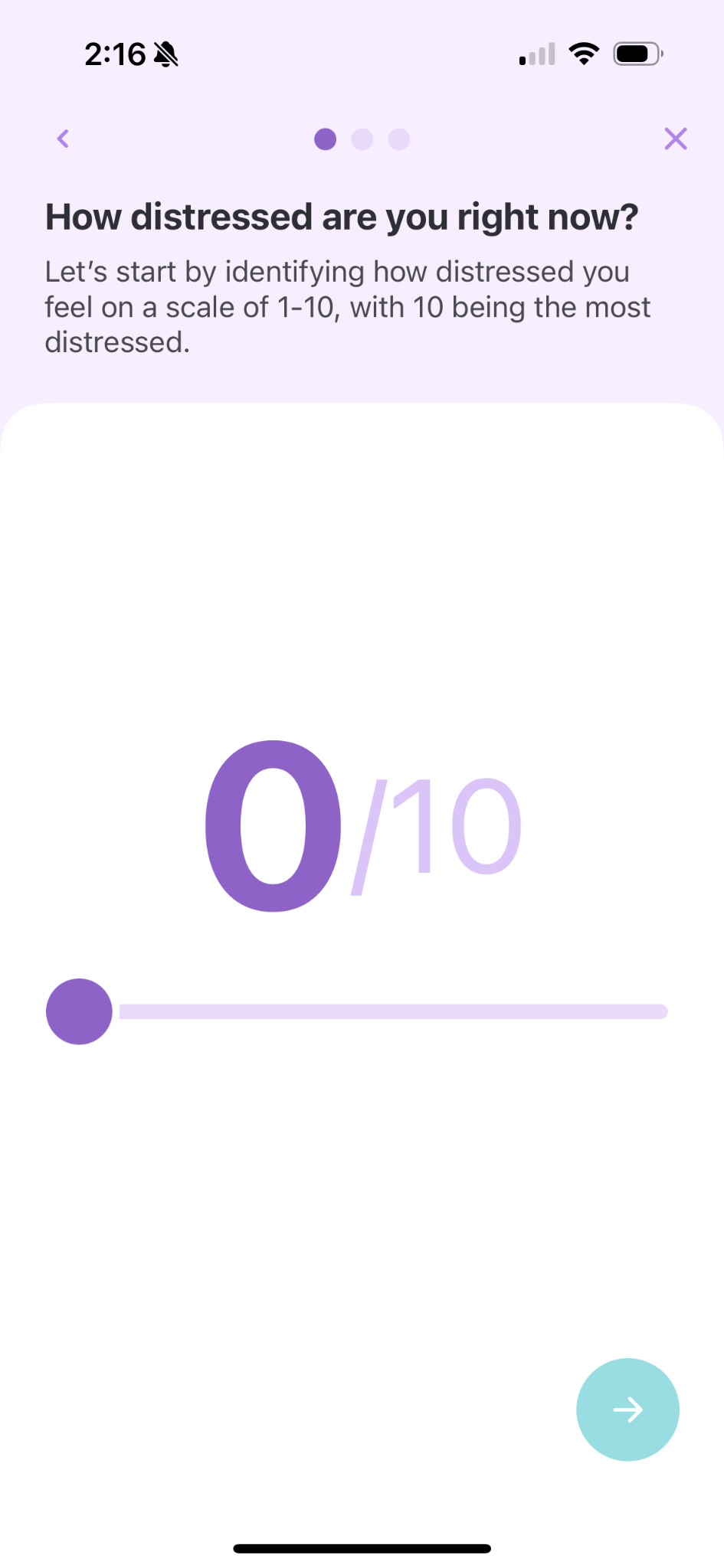

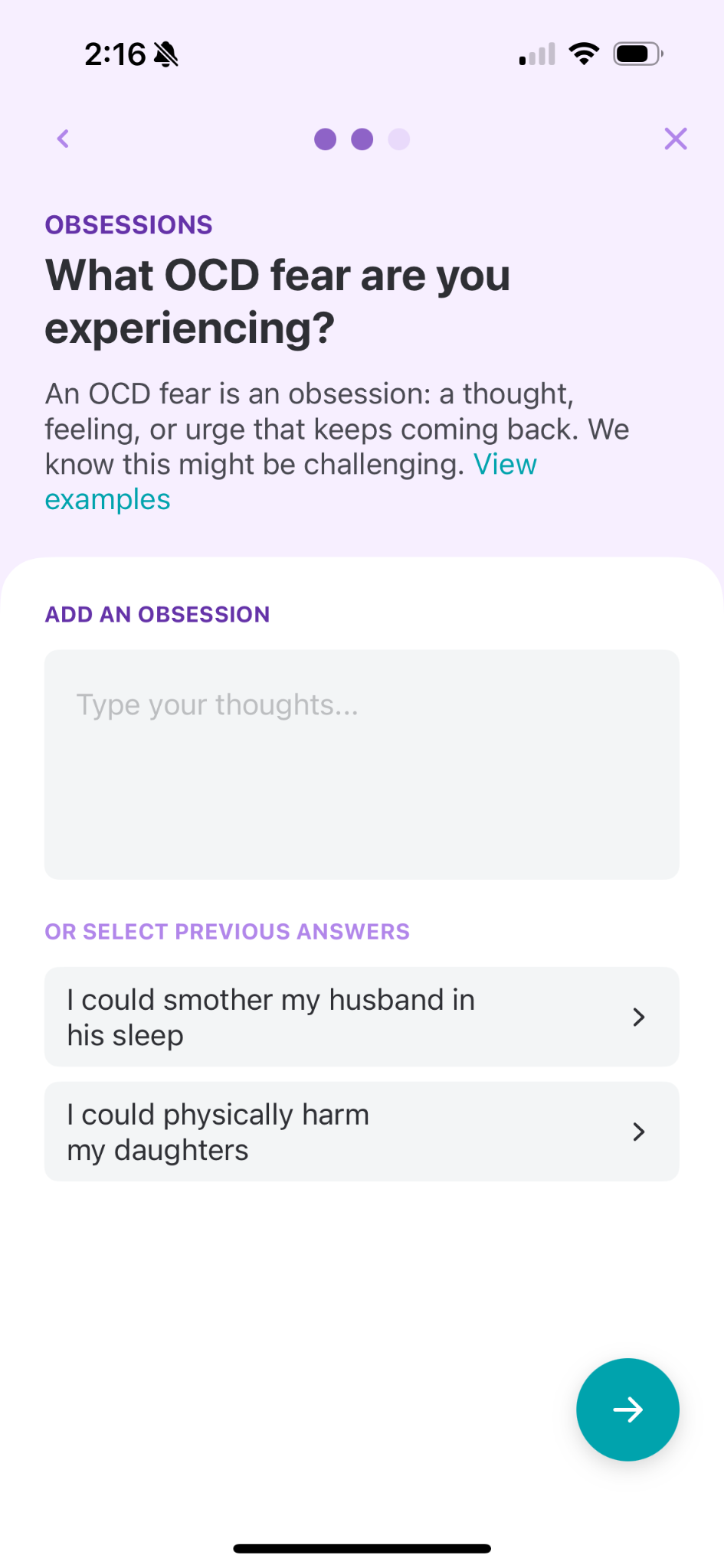

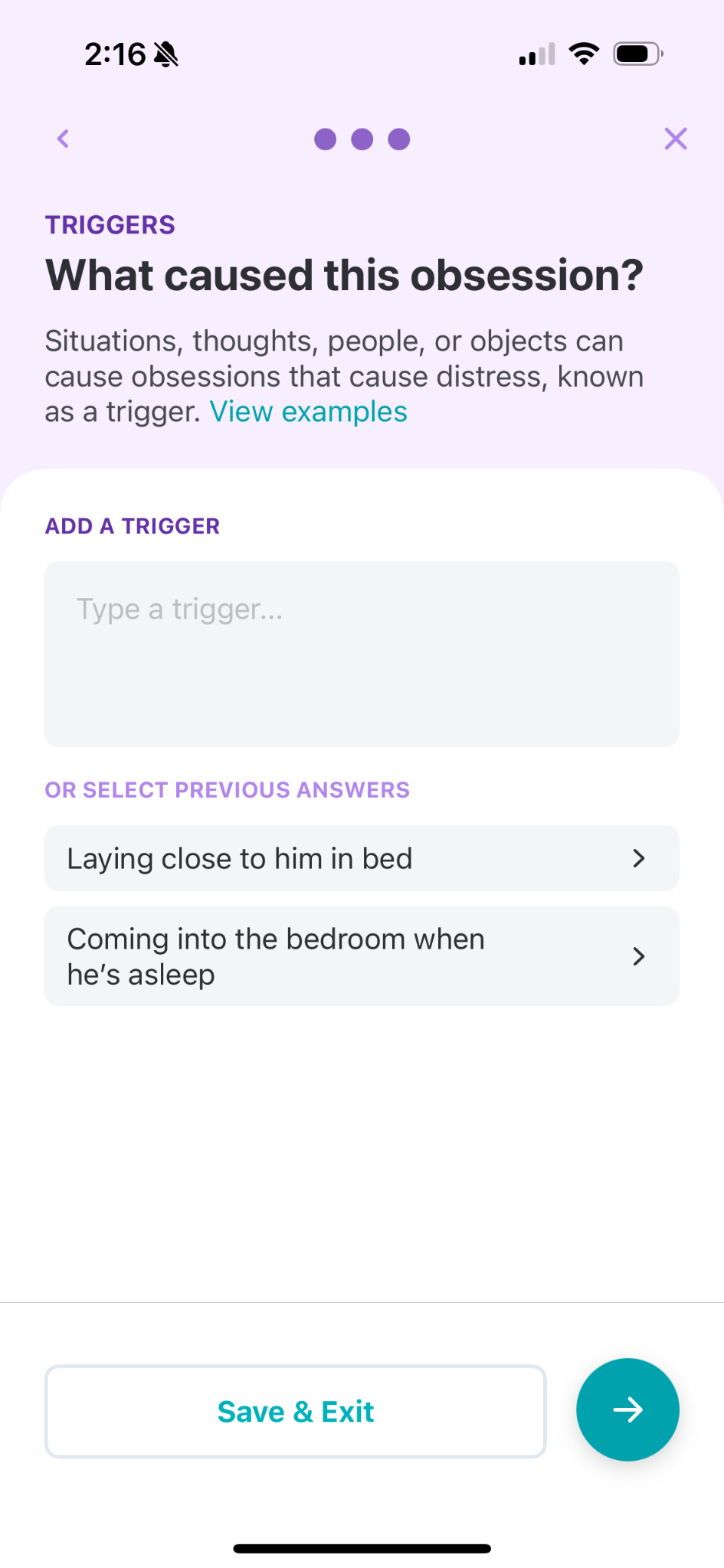

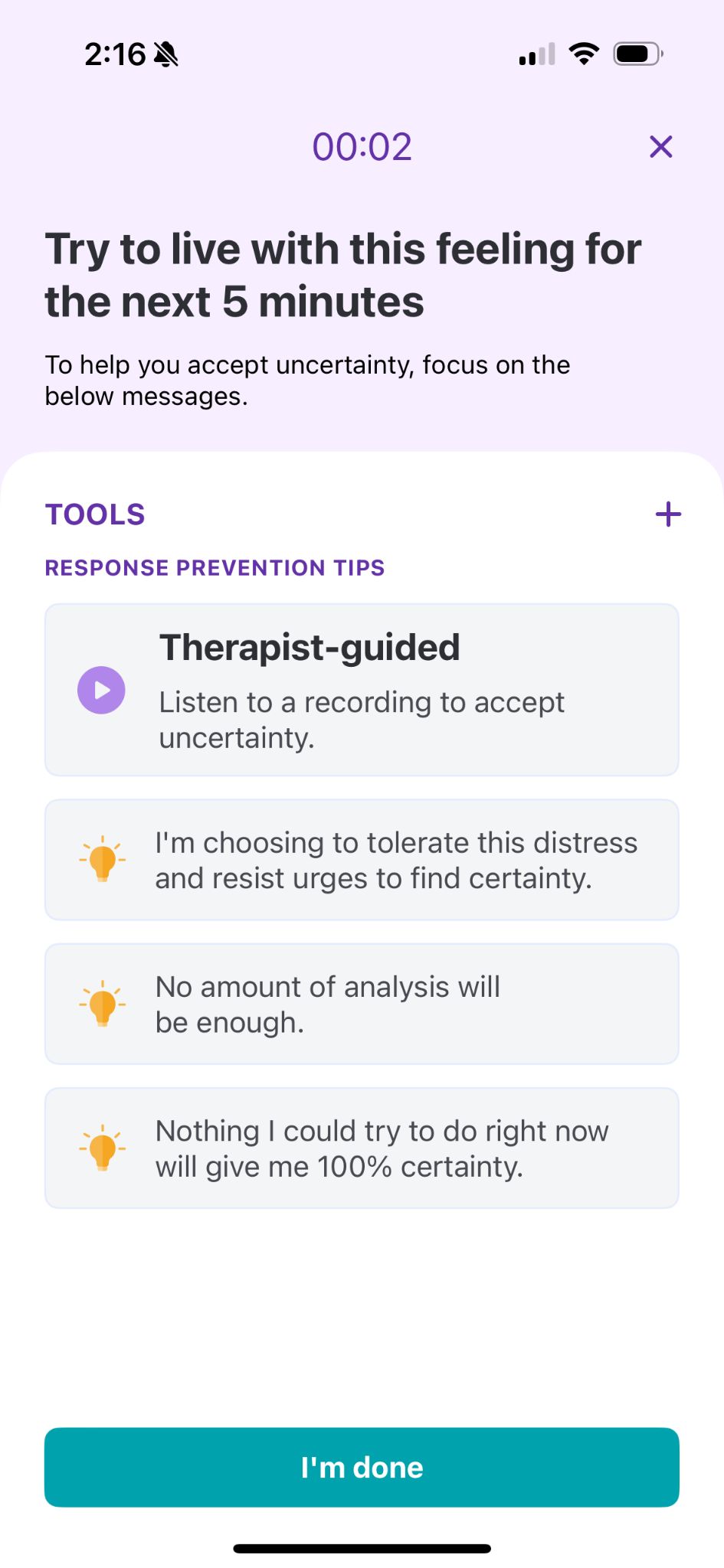


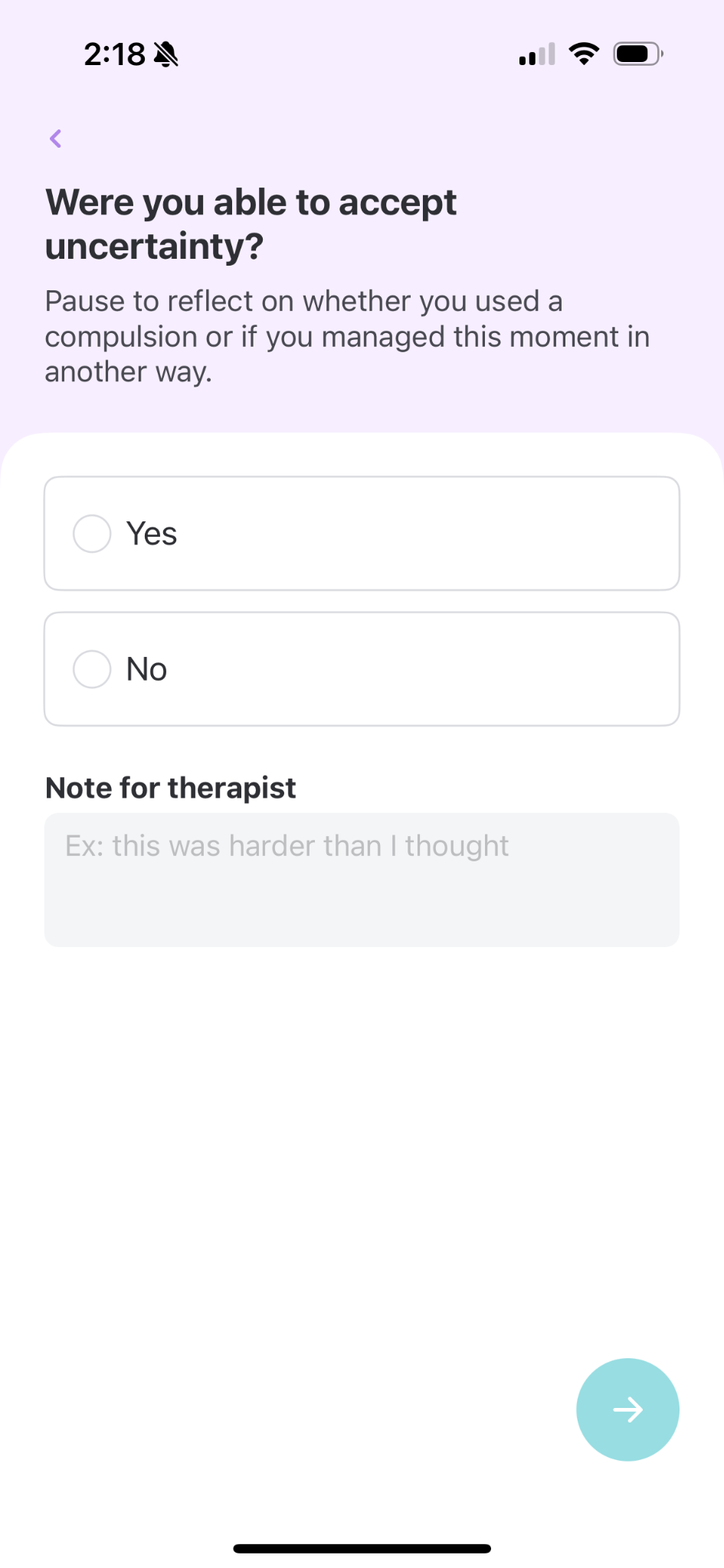

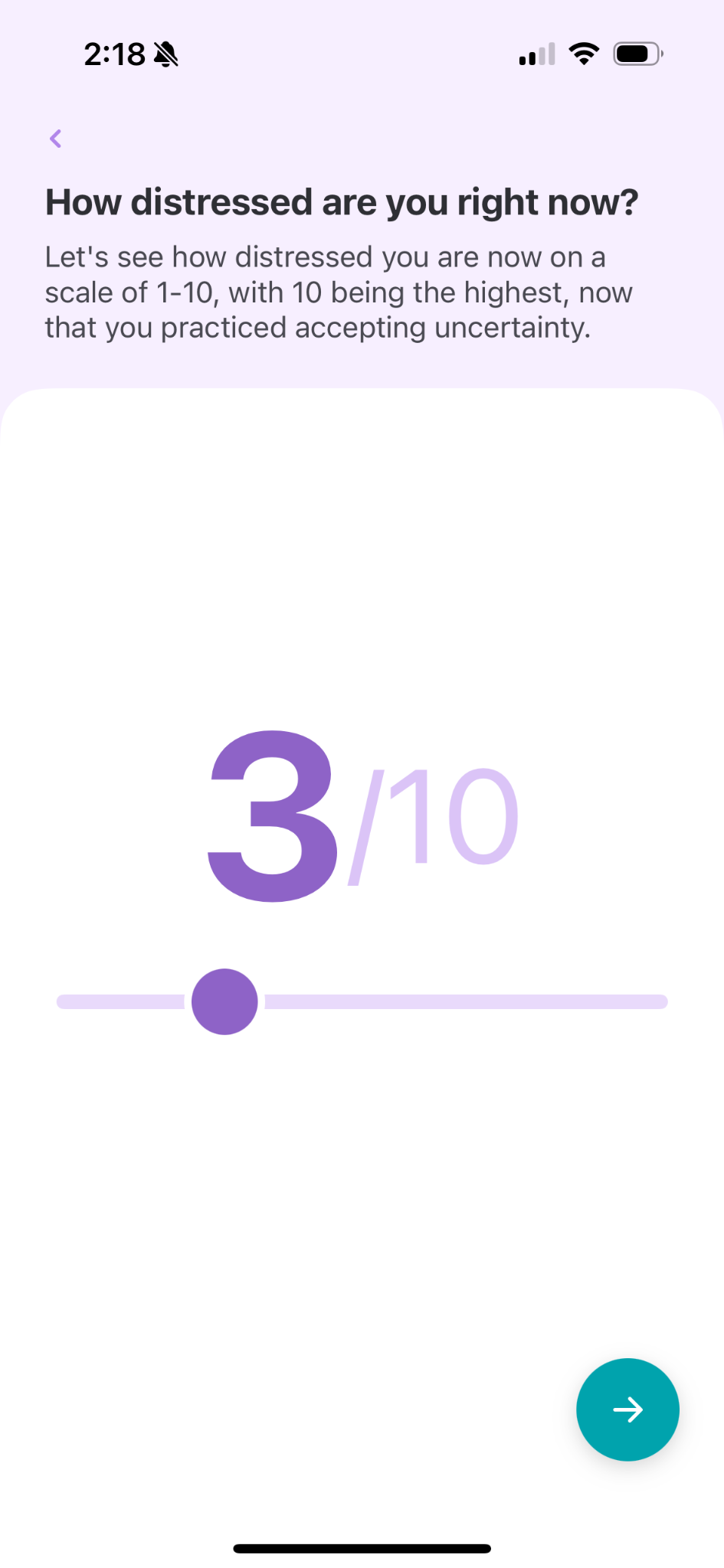

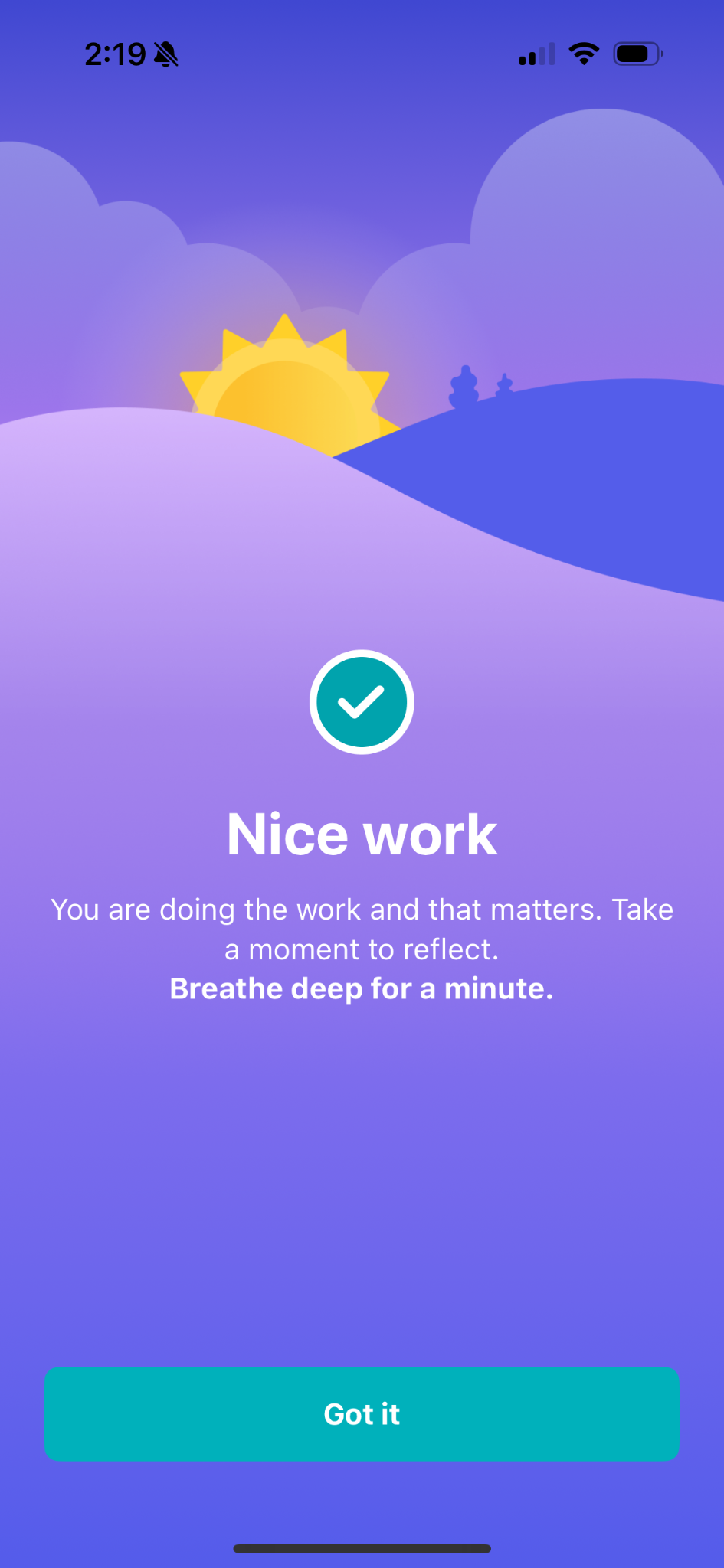

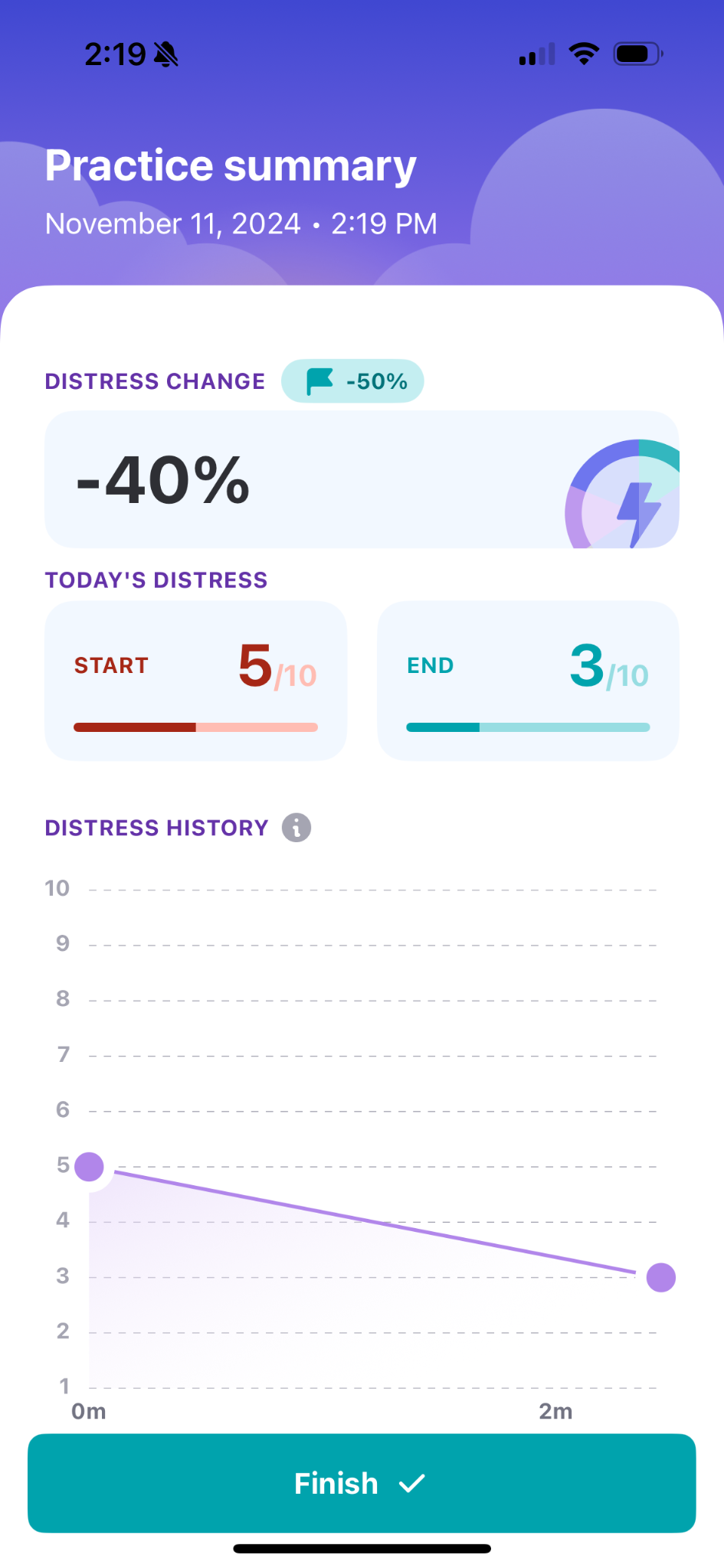


Fig. 5. NOCD App SOS feature
